# A Viscosity-Based Model for Bubble-Propelled Catalytic Micromotors

**DOI:** 10.3390/mi8070198

**Published:** 2017-06-23

**Authors:** Zhen Wang, Qingjia Chi, Lisheng Liu, Qiwen Liu, Tao Bai, Qiang Wang

**Affiliations:** 1 Department of Mechanics and Engineering Structure, Wuhan University of Technology, Wuhan 430070, China; wangzhen@whut.edu.cn (Z.W.); qingjia@whut.edu.cn (Q.C.); qiwen_liu@whut.edu.cn (Q.L.); whut_baitao@163.com (T.B.); 2 State Key Laboratory of Advanced Technology for Materials Synthesis and Processing, Wuhan University of Technology, Wuhan 430070, China; 3 Infrastructure Management Department, Wuhan University of Technology, Wuhan 430070, China; qiang_wang@whut.edu.cn

**Keywords:** micromotors, viscosity, mechanical model

## Abstract

Micromotors have shown significant potential for diverse future applications. However, a poor understanding of the propelling mechanism hampers its further applications. In this study, an accurate mechanical model of the micromotor has been proposed by considering the geometric asymmetry and fluid viscosity based on hydrodynamic principles. The results obtained from the proposed model are in a good agreement with the experimental results. The effects of the semi-cone angle on the micromotor are re-analyzed. Furthermore, other geometric parameters, like the length-radius aspect ratio, exert great impact on the velocity. It is also observed that micromotors travel much slower in highly viscous solutions and, hence, viscosity plays an important role.

## 1. Introduction

Micromotors are small devices that can move themselves by converting environmental chemical energy, electrical energy, light energy, and heat energy into kinetic energy when dissolved in a special solution [1]. Due to their high powers to transport cargo and deliver materials to a specific location, micromotors are extensively applied in cell separation, targeted drug delivery, precision nanosurgery, and environmental sustainability [2].

**Figure 1:**
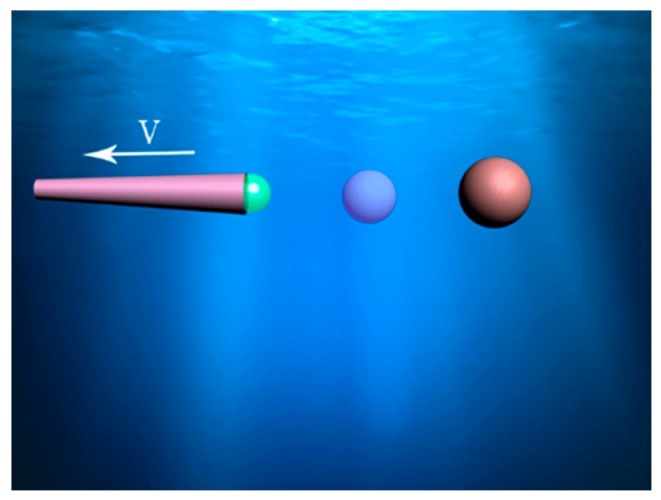
Schematic of the bubble formation and motion of the micromotor.

The possible propulsion mechanisms have been explained as the interfacial tension gradient [3,4], self-electrophoresis [5,6,7], self-diffusiophoresis [8], and micro/nano bubbles [9,10,11,12,13]. The movement of a bubble-propelled micromotor basically depends on catalytic reactions, such as the decomposition of hydrogen peroxide (H_2_O_2_) [5,13,14,15,16] or a Zn-based microtube driven in acidic water [17,18]. Bubble-propelled micromotors driven by chemical reactions are normally analyzed in two shapes: Janus microsphere motor and tubular micromotor. Other geometries like the maple tree fruit or samara are not the commonly used ones [19]. In the experiment, the frequently used tubular micromotors are conical rather than cylindrical. The inner wall of the tube is coated with catalytic metals such as Pt or Zn. The bubbles generated from the inner wall eject one way in the tube, driving the micromotor to move in the opposite direction, as shown in Figure 1. By depositing other functional metals, such as Fe and Ti, onto the outside surface of the tube, such a simple metal layer could be magnetized by an external magnetic field. The magnetic field orients the moving direction without altering the speed. Moreover, the motion can be stopped and initiated by modulating the magnetic field intensity. There are also other ways to control the motion of micromotor, like thermally-driven acceleration and chemical stimuli [20]. This kind of micromotor can be applied in biological medicine, the chemical industry, and environment engineering [20,21,22,23,24,25,26]. 

A large number of studies on bubble-propelled tubular micromotors have been presented to achieve higher efficiency, faster speed, and easier control of motion [11,12,22,27,28,29,30]. Some works mainly focused on the material selection and fabrication technologies of the micromotor [31,32], the reaction process and the control method of the motion [12,32,33]. However, the relationship between the motion of the micromotor and the behavior of the bubble were rarely reported. 

A mass transport model for the bubble was first proposed by Favelukis [9]. Manjare et al. [11] used this model to describe the one-dimensional mass transport and reaction process of cylindrical micromotors by adding a reaction-diffusion term. In [11], the authors predicted the average speed of the micromotor by considering the bubble-growing force as the driving force. The results show that the velocity of the micromotor depends on the length, the opening radius of the cylindrical microjet, and the concentration of the H_2_O_2_ solution. 

Fomin et al. [28] also considered the bubble-growing force in the tube as the crucial driving force. In their work, the bubble that nucleates at the inner wall of the tube grows with chemical reactions until it is large enough to completely touch the internal wall of the tube. At this time, a ‘capillary force’ occurs. The fluid in the tube is separated into two parts by the bubble, generating a net moment. This net moment drives the tube to move forward.

Li et al. [28] used a two-dimensional model for bubble growth and ejection to analyze the velocity of the tubular micromotor. The geometric asymmetry of the bubble caused by the buoyancy force was studied. The experimental and simulation results showed that the velocity of the conical tubular micromotor strongly depends on the semi-cone angle, expelling frequency, and bubble radius ratio. Their conclusions are consistent with the related results [31,32,33,34,35].

The viscosity of fluid plays a vital part in the drag force, particularly when the Reynolds number is low [36]. This will severely affect the model accuracy for predicting the velocity of micromotors [36]. Manjare et al. [37] first proposed fitting results with instantaneous speed and time, and viscosity was taken into account. However, the implications of the relationship between velocity and viscosity were not thoroughly investigated in the previous studies. In the present paper, a mechanical model for micromotors is proposed**.** The model takes into account of the effects of fluid viscosity, H_2_O_2_ concentration, and shape parameters of micromotors. After verification of the published test results, the model is used to analyze the effects of the semi-cone angle, length radius ratio, and viscosity.

## 2. Materials and Methods 

**Figure 2:**
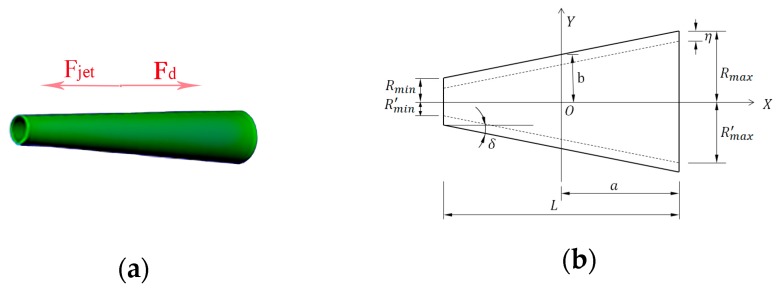
Schematic of the geometry size and force acting on micromotor: (**a**) *F_jet_* and *F_d_* are the driving and drag force caused by bubbles and fluid separately; and (**b**) geometric size of the micromotor model.

The growth and departure of bubbles from a tubular wall is a complex dynamic process due to the momentum and energy transfer between the bubble and the surrounding liquid. To simplify the analysis, a model for tubular micromotor is proposed, as shown in Figure 2. The conical tube has a length of *L* and a wall thickness *η*, with a larger end opening radius *R_max_* and a semi-cone angle *δ*. 

The internal surface of the tube is coated with layer of catalyst-platinum (Pt). When immersed in H_2_O_2_ solution, the bubble nucleates from the decomposition of H_2_O_2_ into O_2_ due to the Pt layer. The net surface tension force drives the bubble to move to the larger opening of the tube. When the bubble reaches the larger end, it ejects or bursts, which generates a force to push the micromotor forward. This driving force is shown in Figure 2a. 

According to Klausner et al. [38], $F_{jet}$ can be expressed as:

(1) $$F_{jet}/6\pi \mu v_{b}R_{b}=\frac{2}{3}+{\left[{\left(\frac{12}{Re}\right)}^{n}+0.796^{n}\right]}^{-1/n}$$

As it has already mentioned above, if the Reynolds number is low here, the last term of the equation can be neglected. Thus, the expression for the driving force on a moving micromotor is:

(2) $$F_{jet}=\frac{2}{3}\cdot 6\pi \mu v_{b}R_{b}=4\pi \mu v_{b}R_{b}$$

where *μ* is the fluid viscosity of H_2_O_2_ solution; $v_{b}$ and $R_{b}$ are the velocity and radius of the bubble. The velocity of bubble is not easy to measure, hence, the oxygen productivity *q* is here:

(3) $$q=v_{b}A_{b}=v_{b}\pi R_{b}^{2}$$

By combining Equations (2) and (3), the driving force can be expressed as:

(4) $$F_{jet}=\frac{4\mu q}{R_{b}}$$

where oxygen productivity *q* is proportional to the inner area of Pt surface *S* and H_2_O_2_ concentration *CH_2_O_2_*, so *q* can be expressed as [27]:

(5) $$q=nC_{H_{2}O_{2}}S$$

(6) $$S=\frac{\pi L}{cos\delta }\left(R_{max}\prime +R_{min}\prime \right)=\frac{\pi L}{cos\delta }\left(2R_{max}\prime -Ltan\delta \right)$$

The parameter *n* is 7.35 × 10^−4^ m/s with reference to the experiment data [34].

*F_d_* is the drag force caused by fluid environment. For a conical micromotor, *F_d_* can approximately be expressed as [39]:

(7) $$F_{d}=\frac{2\pi \mu Lv}{ln\left(L/b\right)+C_{1}}$$

where *C_1_* is the shape coefficient:

(8) $$\begin{array}{ll} C_{1}=-\frac{1}{2}+ln2 & \\ & -\frac{2-\xi \;tan\delta }{2\xi \;tan\delta }[\frac{2}{2-\xi \;tan\delta }ln\left(\frac{2}{2-\xi \;tan\delta }\right)\\ & -\frac{2-2\xi \;tan\delta }{2-\xi \;tan\delta }ln\left(\frac{2-2\xi \;tan\delta }{2-\xi \;tan\delta }\right)] \end{array}$$

and *ξ* is the length radius aspect ratio, $\xi =L/R_{max}$.

The equation of motion for the micromotor under this condition may be developed as:

(9) $$m\dot{v}=\sum F=F_{jet}-F_{d}$$

Here, *m* is the mass of the micromotor; this parameter depends on the density and volume of the micromotor. The volume *V_j_* is calculated based on the geometry:

(10) $$\begin{array}{ll} m=\rho V_{j}= & \rho [\frac{\pi }{3}L\left({R_{max}}^{2}+R_{max}R_{min}+{R_{min}}^{2}\right)\\ & \;-\frac{\pi }{3}L\left(R_{max}\prime^{2}+R_{max}\prime R_{min}\prime +{R_{min}}^{\prime 2}\right)] \end{array}$$

Combining Equations (6) with (1)–(3), it can be turned into:

(11) $$m\dot{v}+\frac{2\pi \mu L}{ln\left(L/b\right)+C_{1}}v=\frac{4\mu q}{R_{b}}$$

*R_b_* in this equation is a time-dependent parameter and can be expressed in terms of the volume of the bubble, as follows:

(12) $$R_{b}={\left(\frac{3V_{b}}{4\pi }\right)}^{\frac{1}{3}}={\left(\frac{3qt}{4\pi }\right)}^{\frac{1}{3}}$$

Therefore, the differential equation to be solved can be written as:

(13) $$m\dot{v}+\frac{2\pi \mu L}{ln\left(L/b\right)+C_{1}}v=4\mu q{\left(\frac{3qt}{4\pi }\right)}^{-\frac{1}{3}}$$

The differential equation can be analytically integrated for an initial velocity *v_0_* (infinitely tending to zero). The instantaneous velocity of micromotor is shown below:

(14) $$v=\frac{4\mu q}{m}{\left(\frac{3q}{4\pi }\right)}^{-\frac{1}{3}}K+v_{0}e^{-At}$$

Thus, the average velocity of the micromotor can be expressed as:

(15) $$\bar{V}=\frac{1}{t}\int_{0}^{t}v\left(\tau \right)d\tau$$

The parameter *A* is given by:

(16) $$A=\frac{2\pi \mu L}{m\left(ln\left(L/b\right)+C_{1}\right)}$$

*K* is obtained from the complicated time integration:

(17) $$K=e^{-At}\int t^{-\frac{1}{3}}e^{-At}dt$$

Expressing *K* in terms of the Gamma function, *K* takes the form:

(18) $$K=e^{-At}\int t^{-\frac{1}{3}}e^{-At}dt=\overset{n}{\underset{i=1}{\sum }}{\left(-1\right)}^{i+1}\frac{\Gamma \left(-\frac{1}{3}+1\right)}{\Gamma \left(-\frac{1}{3}+2-i\right)A^{i}}t^{-\frac{1}{3}+1-i}$$

Thus, the parameter *K* may be expressed in the series form:

(19) $$K=A^{-1}t^{-\frac{1}{3}}+\frac{1}{3}A^{-2}t^{-\frac{4}{3}}+\frac{4}{9}A^{-3}t^{-\frac{7}{3}}+\frac{28}{27}A^{-4}t^{-\frac{10}{3}}+\ldots$$

Based on Equation (15), in order to solve this equation, we use Fortran to do the integral operation. The velocity of the micromotor depends on the following factors: H_2_O_2_ concentration *CH_2_O_2_*, geometric parameters of the micromotor, like *L*, *R_max_*, and *δ*, the mass of the micromotor *m*, and the fluid viscosity *μ*. The influence of these factors will be discussed in detail.

The calculation of this work is much simpler than that of Li et al. [27], regardless of the complicated processes of bubble nucleation, growth, departure, ejection, and burst. The contribution of these processes is treated as ‘the driving force’ for the micromotor to move forward. The consistency between the theoretical results and experimental results confirms that the accuracy of the calculation is acceptable.

## 3. Results and Discussion

### 3.1. Concentration of H_2_O_2_

**Figure 3:**
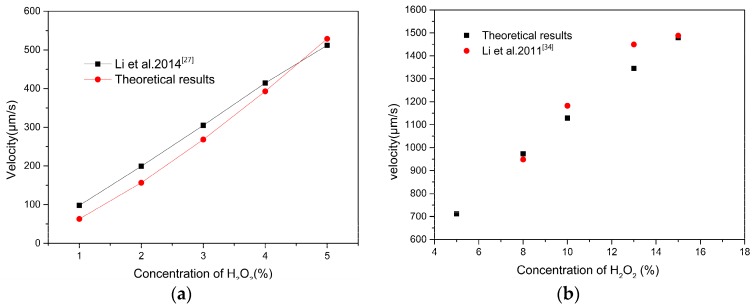
The velocity of micromotor versus H_2_O_2_ concentration: (**a**) at low concentration (<5%) with length *L* = 9.1 μm, $R_{max}$ = 1.16 μm, *δ* = 2.3°, *CH_2_O_2_* = 1–5%; and (**b**) at high concentration (>5%) with length *L* = 100 μm, $R_{max}$ = 10 μm, *δ* = 0°, *CH_2_O_2_* = 5–15%.

The dependence of the velocity on H_2_O_2_ concentration is listed below. Micromotors with two different shapes are immersed in solutions with two different concentrations. One with length *L* = 9.1 μm, $R_{max}$ = 1.16 μm, *δ* = 2.3° immersed in H_2_O_2_ concentrations ranging from 1% to 5% and the other with length *L* = 100 μm, $R_{max}$ = 10 μm, *δ* = 0° immersed in H_2_O_2_ concentrations ranging from 5% to 15%. The results obtained based on Equation (15) are shown in Figure 3. The speed increases rapidly with increasing solution concentration.

Figure 3a shows the comparison between our result and the experimental results obtained from Li et al. [27]. The results show a good agreement with experimental data. For further illustration, another cylindrical model was developed to simulate high H_2_O_2_ concentrations, as shown in Figure 3b. Results from our study are happened to be in good agreement with Li et al. [34]. A well-defined propulsion is observed over a higher peroxide concentration, with speed ranging from 62.7 μm/s to 1480 μm/s, when the H_2_O_2_ concentrations are 1% (conical tube, inset a) and 15% (cylindrical tube, inset b), respectively. Raising the H_2_O_2_ concentration increases the catalytic reactivity toward the decomposition of hydrogen peroxide. The driving force in Equation (4) strongly depends on oxygen gas productivity *q*. The propel efficiency is improved by the increase of the H_2_O_2_ concentration. Thus, Equation (15) can provide a good prediction to the motion of the micromotor.

### 3.2. Semi-Cone Angle

**Figure 4:**
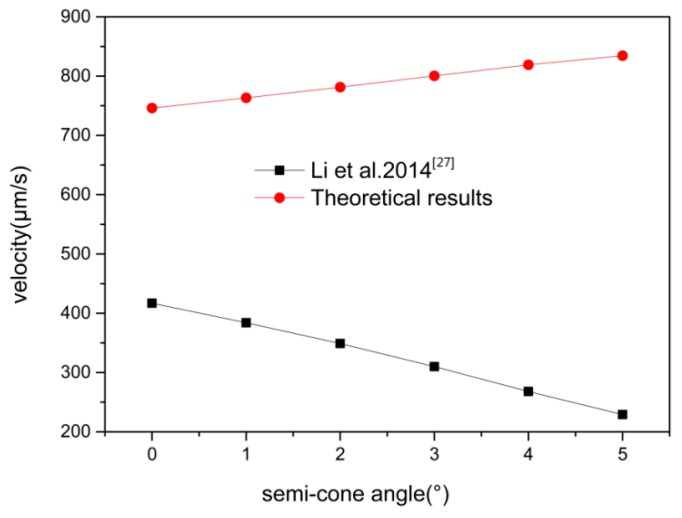
The velocity versus the semi-cone angle of the micromotor, the model used here is length *L* = 100 μm, $R_{max}$ = 10 μm, *CH_2_O_2_* = 5%, *δ* = 0°–5°.

Our results about the semi-cone angle stirred a small deviation as compared with Li et al. [27]. In Figure 4, Li et al. [27] presented that the average velocity decreased with an increase in the semi-cone angle for the same *R_max_* and *L*. 

Results gained in this paper show that the velocity increased with the increase of the semi-cone angle. This was verified by recent experimental styudies carried out by Fomin et al. In Fomin’s theory [28], as a result of the geometric asymmetry for a tubular micromotor (conical), a capillary force occurs and tends to push bubbles towards the larger opening of the tube. A driving force is generated on the tube in the opposite direction and causes the micromotor to move forward. For the symmetric shape of the micromotor (cylindrical), the bubbles may move to either opening of the tube at initial time, which influence the dynamics and lower the average velocity.

Li et al. [40] neglect the effect of bubble moving inside the tube on the motion of micromotor, and assume that the driving force comes from the bubble growth and ejection at the end of tube. They also studied different shapes of micromotors, and their result revealed that the drag forces decreased with an increase of the semi-cone angle with the same *R_max_* and length *L*. In order to find out the hydrodynamic behavior of two different shapes, a numerical simulation using the commercial software package Fluent 18.0 (ANSYS) has been carried out.

**Figure 5:**
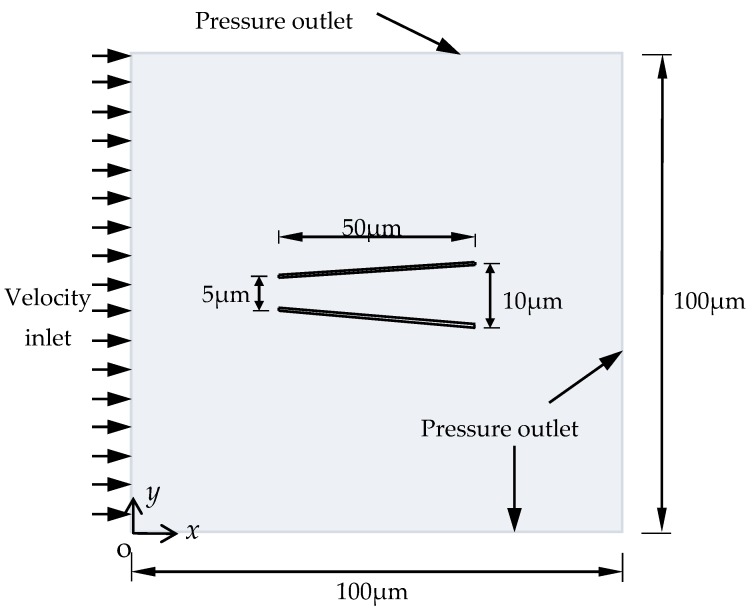
The boundary conditions and the shape of the micromotor.

Micromotos of the two shapes are immersed in the same surrounding conditions with density ρ = 998.2 kg/m^3^ and viscosity *μ* = 1.003 mPa∙s. Since the fluid area, the micromotor, and the boundary conditions are all symmetric, a two-dimensional model was set up by using Fluent in Figure 5. A model of the domain fluid area 100 μm × 100 μm was built. On the left side, there is a velocity inlet boundary condition (100 μm/s). The other three sides are pressure outlet boundary conditions (Figure 5), and the static pressure at the outlet boundary is 0. The two different micromotors were immersed in the center of the fluid: the cylindrical one with length *L* = 50 μm, radius *R* = 5 μm, and wall thickness *η* = 1 μm; the conical one with length *L* = 50 μm, *R_min_* = 5 μm, *R_max_*= 10 μm, and wall thickness *η* = 1 μm (Figure 5). The no-slip boundary condition is enforced at the walls around the micromotor.

The results show there is a spontaneous pressure difference between the two openings of the tube and that causes the bubble to move from one end to another.

**Figure 6:**
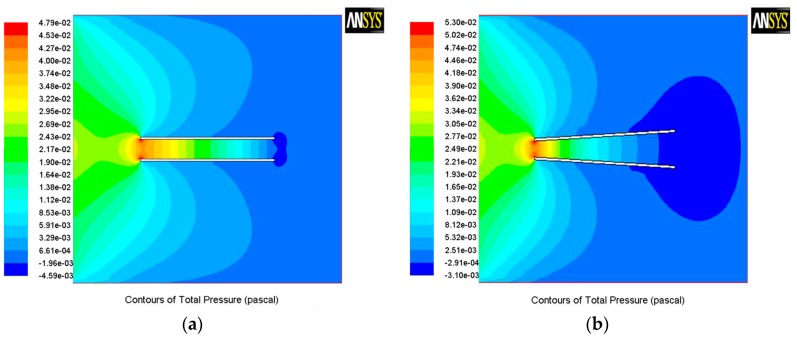
The pressure contours of two differently shaped micromotors with all of the same boundary conditions: (**a**) cylindrical micromotor; and (**b**) conical micromotor.

Figure 6 shows the pressure contours of a cylindrical tube (inset a) and a conical one (inset b). The pressure differences between the two openings in the conical tube are much larger than the cylindrical one, which means bubbles in the conical tube move more easily from one end to the other.

Other interpretations are similar to the above. Bubbles in the conical tube are easier to move to the larger end due to the net surface tension force [38]. In our theory, the conical tube exhibits a larger area of the contact surface than the cylindrical one. This means the q of the conical tube is larger than that of the cylindrical tube, as is evident from Equation (5). The driving force is then enhanced, while the drag force remains unchanged; thus, the velocity increases.

### 3.3. Length-Radius Aspect Ratio

**Figure 7:**
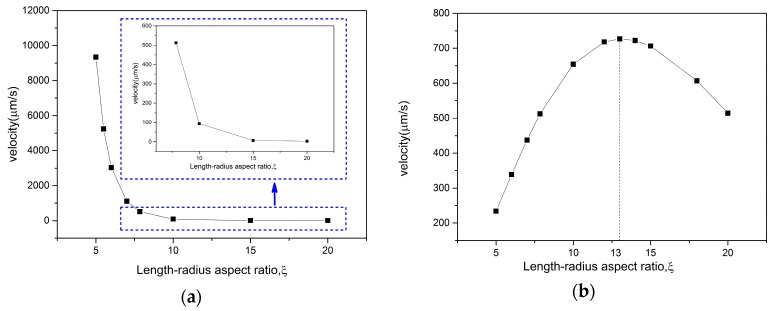
The velocity versus length-radius aspect ratio: (**a**) change the radius $R_{max}$, keeping L=9.1 μm constant; and (**b**) change the length L, keeping $R_{max}=1.16$ μm constant.

Figure 7 below illustrates the effects of length-radius aspect ratio *ξ* on the velocity for different shapes of micromotors. Using the definition of the length-radius aspect ratio $\xi =L/R_{max}$, the parameter *ξ* can be calculated in two ways, namely varying $R_{max}$ when fixing *L*, or varying *L* when fixing $R_{max}$. The model used in this section was from Figure 3a: with a semi-cone angle *δ* = 2.3° and the concentration of solution *CH_2_O_2_* = 5%. Figure 7a shows that the velocity decreases when *ξ* increased from 5 to 19 (keeping *L* = 9.1 μm unchanged, the radius $R_{max}$ varies from 1.82 μm to 0.479 μm), the velocity is especially high when *ξ* is around 5. There are two major influencing factors: the drag force and inner surface area.

The drag force used in Equation (7) is a simplified form for a spheroid with semi-axes *a* and *b* in Figure 2b. Its original form on the ellipsoid is [39]:

(20) $$F_{d}=16\pi \mu bv{\left\{-\frac{2\left(a/b\right)}{{\left(a/b\right)}^{2}-1}+\frac{2{\left(a/b\right)}^{2}-1}{{\left[{\left(a/b\right)}^{2}-1\right]}^{\frac{3}{2}}}ln\left[\frac{a/b+{\left({\left(a/b\right)}^{2}-1\right)}^{\frac{1}{2}}}{a/b-{\left({\left(a/b\right)}^{2}-1\right)}^{\frac{1}{2}}}\right]\right\}}^{-1}$$

while b/a can be described as:

(21) $$\frac{b}{a}=\frac{\frac{R_{max}+R_{min}}{2}}{\frac{L}{2}}=\frac{R_{max}+R_{min}}{L}$$

**Figure 8:**
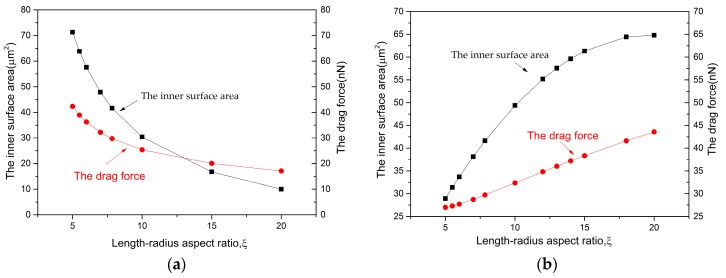
The inner surface area and drag force versus length-radius aspect ratio ξ: (**a**) change the radius $R_{max}$, keeping *L* = 9.1 μm constant; and (**b**) change the length *L*, keeping $R_{max}$ = 1.16 μm constant.

Only when b/a→0, Equation (20) would be reduced to Equation (7). So, taking $\xi =L/R_{max}=5$ as an example, the value of $F_{d}$ is 42 nN and 12 nN when using Equations (7) and (20), separately. The drag force is overestimated when *ξ* is small. Another influencing factor is the inner surface area, which is closely related to the oxygen productivity *q*. When the parameter *ξ* is increased from 5 to 19, the inner surface area decreased from 79 μm^2^ to 2 μm^2^. The larger surface brings higher oxygen productivity, which produces a stronger driving force in return. Both the drag force and inner surface area decrease with the increase of *ξ* (Figure 8a). Synthesizing both factors, it shows that surface area displays greater influence than the drag force. This is the reason why velocity decreases when the length-radius aspect ratio *ξ* increases.

The other option is to change the length *L* from 5.8 μm to 22.04 μm, keeping $R_{max}=1.16$ μm constant. So, *ξ* varies from 5 to 19, too. The relationship between velocity and length-radius aspect ratio *ξ* shows a threshold in Figure 7b based on Equation (15). Figure 8b shows how the two influencing factors change with a series of *ξ*. The increasing area promotes the motion of micromotor, i.e., increasing the velocity. Meanwhile, an increase in drag force hinders the motion, i.e., decreasing the velocity. The increment of surface area becomes smaller along with the increasing *ξ*. As it has been mentioned before, the surface area displays greater influence than the drag force. Hence, they neutralized with each other at the point of *ξ* = 13, as shown in Figure 7b.

There exists an optimal length *L* = 15.08 μm when keeping the radius $R_{max}$ = 1.16 μm unchanged (*ξ* = 13). This indicates that, for each radius, there exists an optimal length. The length of the micromotor should be neither too long nor too short. On the other hand, there would be no optimal radius when keeping the length unchanged. Theoretically speaking, the larger radius of the tube is the better since a larger inner surface produces more gases. However, in practice, there is no such shaped micromotor in biomedical or industrial areas due to the fact that the drag force would be extremely large and the motion control of such a micromotor is more difficult. Gao et al. [41] obtained different speeds through experiments and attributed it to the size of the opening diameter. Thus, the discussion above introduces an optimized design method for the structural shape to obtain more efficient microengines and holds considerable promise for diverse future applications.

### 3.4. Fluid Viscosity

The theoretical framework developed in this paper is not only suitable for the catalytic micromotor by dissolution of H_2_O_2_, but also for self-reacted or other micromotors. We extend the discussion of the foregoing results to other factors like fluid viscosity. In this part, 24 different viscosities have been calculated based on Equation (15). According to our results, the velocity decreases dramatically along with the increasing of viscosity from 0.1 mPa·s to 4 mPa·s. The results also show that there is a non-linear relationship below 1.5 mPa·s and nearly linear above 1.5 mPa·s. The results show good agreement with the experimental results from Li et al. [42]. This indicates that the velocity is rather sensitive to fluid viscosity.

**Figure 9:**
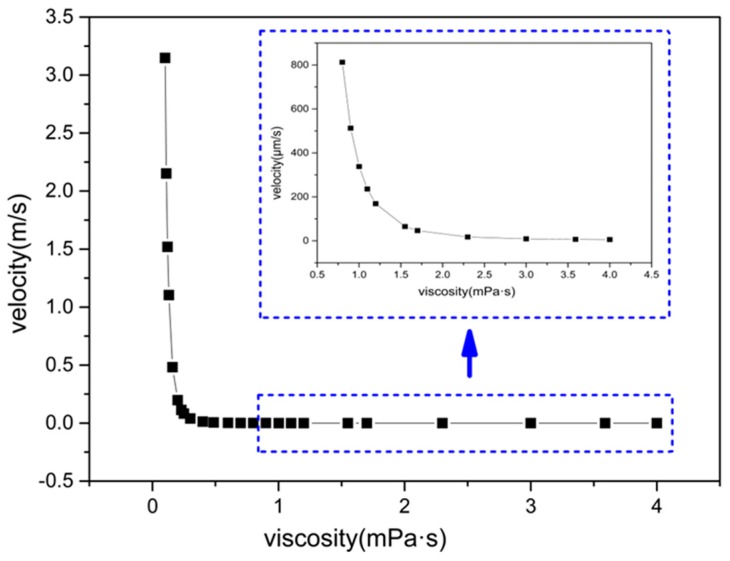
The velocity versus viscosity of fluid.

As is shown in Figure 9, the velocity drops by 93.71% when the viscosity increases form 0.1 mPa·s to 0.2 mPa·s. When the viscosity continues to increase, the descending rate of velocity slows. Thus, in order to improve the micromotor’s velocity, one way is to reduce the fluid viscosity as low as possible when the viscosity is less than 0.2 mPa·s. If the viscosity is larger than 0.2 mPa·s, the velocity will not change so rapidly when increasing the viscosity. The inset in Figure 9 shows even when the viscosity is larger than 1 mPa·s, the speed of the micromotor still decreases upon increasing the viscosity of the solution. The velocity of the micromotor reaches 513 μm/s when the viscosity of H_2_O_2_ is 0.9 mPa·s, but the velocity is only 5 μm/s when the viscosity is 4 mPa·s irrespective of the chemical reaction that may occur. A slight change in viscosity brings a significant decrease/increase in velocity. Gao et al. [29,31] reported the observation regarding motion of catalytic micro/nano motors in biological environments, such as cell culture media and human serum. The speed exhibits a reduction by 50% and 68%, respectively, than the speed in water under similar conditions. The decrease is attributed to high viscosity, as well as passivation of the catalytic Pt surface. As far as our knowledge is concerned, there are very few investigations referring to the effect of viscosity, especially experimental results. 

The mass of a micromotor also influences the motion behavior. From Equation (10), it is known that the mass changes correspondingly with the shape. This influence factor (length, radius, and semi-cone angle, etc.) has already been discussed above. Only when the shape of the micromotor is fixed, the volume remains constant. The fabricating material chosen for the micromotor should be as light as possible to maintain good performance.

A number of in vivo studies on artificial micromotors have been reported recently. They exhibit many improvements compared to native bio-motors, such as high and controllable velocity, cargo-carrying power, and long lifetime. Nonetheless, accurate mechanical models of artificial micromotors are in high demand to improve the understanding of the dynamic behavior. It is well known that the viscosity in biological environments is considerably larger than 0.2 mPa·s, so the application of micromotors in vivo will be less efficient. Our work offers a clear correlation between micromotor velocity and fluid viscosity and can aid in the design of synthetic micromotors for specific applications.

## 4. Conclusions

In conclusion, we have proposed a new mechanical model to describe the translational motion of micromotors. The motion behavior in solution is analyzed systematically as a function of hydrogen peroxide concentration, geometric parameters and fluid viscosity. All parameters influence the velocity of the micromotor. The contribution from a complex process of bubble nucleation, growth, departure, ejection, and burst is lumped into a reference force when evaluating the driving force, as shown in Equation (4). A comparison between the proposed model and those from the literature has been made and the results showed they are in good agreement. Detailed analyses of various influencing factors have been presented. It was found that the velocity of the micromotor increased approximately linearly with the concentration of hydrogen peroxide. One of the geometric parameters, the semi-cone angle of micromotor, also has the same linear characteristic. Other geometric parameters, like the length-radius aspect ratio, directly influence the locomotion of the micromotor in two ways: on one hand, when fixing length *L*, increasing *ξ* means decreasing the radius $R_{max}$, and the velocity of micromotor decreases dramatically. On the other hand, when fixing radius $R_{max}$, increasing *ξ* means increasing length *L*, and the velocity achieves a threshold. In other words, there exists an optimal length for a certain radius, but there is no optimal radius for a certain length. The fluid viscosity strongly influences the velocity of the catalytic micromotor: when the viscosity increases slightly, the velocity decreases sharply. This work provides deeper insight into the viscosity sensitivity of the micromotor and the model is useful in designing micromotors for biomedical applications. It is expected that this model can ultimately be used to construct versatile and efficient synthetic tubular micro/nanomotors in many areas.
